# Addendum: Eom, T. K. et al. *Dendropanax morbifera* Leaf Polyphenolic Compounds: Optimal Extraction Using the Response Surface Method and Their Protective Effects against Alcohol-Induced Liver Damage. *Antioxidants* 2020, *9*, 120

**DOI:** 10.3390/antiox9090770

**Published:** 2020-08-20

**Authors:** Taekil Eom, Kyeoung Cheol Kim, Ju-Sung Kim

**Affiliations:** 1 Subtropical/Tropical Organism Gene Bank, SARI, Jeju National University, Jeju 63243, Korea; taekil7@hanmail.net; 2 Majors in Plant Resource and Environment, College of Agriculture & Life Sciences, SARI, Jeju National University, Jeju 63243, Korea; cheolst@jejunu.ac.kr

The authors wish to make the following corrections to this paper [1]: This research was supported as a project (grant number C0297060) for the Cooperated R&D between Industry, Academy, and Research Institute funded by the Korea Mistry of SMEs and Startups in 2015, and the Basic Science Research Program through the National Research Foundation of Korea (NRF), funded by the Ministry of Education (grant numbers 2016R1A6A1A03012862 and 2018R1D1A1B07045736). This addendum does not cause any changes to the results or conclusions in the original published paper. The authors would like to apologize for any inconvenience caused to the readers by these changes.
